# Modulation of behaviour and virulence of a high alginate expressing *Pseudomonas aeruginosa* strain from cystic fibrosis by oral commensal bacterium *Streptococcus anginosus*

**DOI:** 10.1371/journal.pone.0173741

**Published:** 2017-03-16

**Authors:** Richard D. Waite, Muhammad R. Qureshi, Robert A. Whiley

**Affiliations:** 1 Centre for Immunobiology, Blizard Institute, Barts and The London School of Medicine and Dentistry, Queen Mary University of London, London, United Kingdom; 2 Centre for Clinical and Diagnostic Oral Sciences, Institute of Dentistry, Barts and The London School of Medicine and Dentistry, Queen Mary University of London, London, United Kingdom; Universiteit Gent, BELGIUM

## Abstract

Cystic fibrosis (CF) airways harbour complex and dynamic polymicrobial communities that include many oral bacteria. Despite increased knowledge of CF airway microbiomes the interaction between established CF pathogens and other resident microbes and resulting impact on disease progression is poorly understood. Previous studies have demonstrated that oral commensal streptococci of the Anginosus group (AGS) can establish chronic pulmonary infections and become numerically dominant in CF sputa indicating that they play an important role in CF microbiome dynamics. In this study a strain of *Pseudomonas aeruginosa* (DWW2) of the mucoid alginate overproducing phenotype associated with chronic CF airway infection and a strain of the oral commensal AGS species *Streptococcus anginosus* (3a) from CF sputum were investigated for their ability to co-exist and their responses to biofilm co-culture. Bacteria in biofilms were quantified, pyocyanin expression by DWW2 was measured and the effect of AGS strain 3a on reversion of DWW2 to a non-mucoidal phenotype investigated. The virulence of DWW2, 3a and colony variant phenotypes of DWW2 in mono- and co-culture were compared in a *Galleria mellonella* infection model. Co-culture biofilms were formed in normoxic, hypercapnic (10% CO_2_) and anoxic atmospheres with the streptococcus increasing in number in co-culture, indicating that these bacteria would be able to co-exist and thrive within the heterogeneous microenvironments of the CF airway. The streptococcus caused increased pyocyanin expression by DWW2 and colony variants by stimulating reversion of the mucoid phenotype to the high pyocyanin expressing non-mucoid phenotype. The latter was highly virulent in the infection model with greater virulence when in co-culture with the streptococcus. The results of this study demonstrate that the oral commensal *S*. *anginosus* benefits from interaction with *P*. *aeruginosa* of the CF associated mucoid phenotype and modulates the behaviour of the pseudomonad in ways that may be clinically relevant.

## Introduction

In the majority of biotic and abiotic environments bacteria exist in dynamic, multispecies biofilm communities within which cooperative and competitive interactions between members are key to survival [1,2]. On a living host multispecies biofilms occur throughout the body, playing an important role in maintaining health and in disease pathogenesis [3,4,5,6,7]. Our increased awareness of the polymicrobial nature of many diseases has been due in no small way to the availability of culture-independent community analysis methodologies [8,9,10]. In the context of cystic fibrosis (CF) these approaches together with culture-based methods have revealed the complexity of the microflora of the adult CF lung and have broadened the focus of many investigations from traditional approaches centred on CF pathogens such as *Pseudomonas aeruginosa*, the *Burkholderia cepacia* complex and *Stenotrophomonas maltophilia* to wider consideration of the CF microbiome including bacterial species usually disregarded as commensals [11,12,13,14]. Studies have shown the CF lung and airways to be heterogeneous environments supporting a wide range of metabolically diverse bacteria in spatially distributed communities [15,16,17]. The mucus in the CF lung gives rise to steep oxygen gradients resulting in areas that are able to maintain high numbers of facultatively and obligate anaerobic bacteria with the oral cavity and upper respiratory tract thought to be the origin of many of these species [11,13,18,19,20].

In contrast to our knowledge of the microbial diversity in CF infected airways the nature and outcomes of the interactions taking place between members of these communities and the importance of such interactions to patient stability or disease progression is scant indeed [21,22]. One group of bacteria that have attracted attention in this respect are the viridans streptococci that are commensals of the oral cavity and upper respiratory tract and have been detected in the infected airways of over 90% of adult CF patients [23]. Filkins et al. [24] found that increased fractional representation of the genus *Streptococcus* in outpatient CF sputa compared with inpatients was the strongest predictor of patient stability with several of the viridans taxa, notably *Streptococcus salivarius*, *Streptococcus parasanguinis* and the Anginosus (‘*Streptococcus milleri*’) group (AGS), the most prevalent in this patient group. In contrast the studies by Sibley et al [25] and Parkins et al [26] found that AGS frequently established chronic pulmonary infections, were the numerically dominant pathogen at the onset of nearly 40% of acute pulmonary exacerbations and gave a positive correlation between AGS disease and co-colonization with *P*. *aeruginosa* in CF patients through possible interspecies interactions resulting in increased virulence. Gene expression studies have shown that interaction between *P*. *aeruginosa* wound isolate PAO1 and oropharyngeal bacteria including streptococci from CF sputa results in upregulation of genes in *P aeruginosa* that are important for pathogenesis [27].

In our own laboratory *in-vitro* studies examining co-culture biofilm interactions between AGS species and phenotypes of the *P*. *aeruginosa* Liverpool Epidemic strain (LES) demonstrated increased numbers of AGS in co-culture and increased expression of virulence factors by *P*. *aeruginosa* giving a more pathogenic LES+AGS partnership [28]. Similarly LES pathogenicity was potentiated through co-culture with Mitis group streptococci including *Streptococcus oralis*, *Streptococcus mitis*, *Streptococcus gordonii* and *Streptococcus sanguinis* with co-operation or competition between the partners dependent on culture atmospheric conditions and order of colonisation [29]. An important and as yet unexplored area of interest in this context is the interaction between viridans streptococci from CF sputa and *P*. *aeruginosa* expressing the CF associated mucoid phenotype caused by overproduction of the polysaccharide alginate. Mucoidy in *P*.*aeruginosa* arises as a result of adaptation to the environmental stresses of the CF lung and has long been associated with chronic CF lung infection, severity of disease and the occurrence of exacerbations [30,31,32]. Therefore the aim of this study was to determine whether AGS are able to grow in co-culture with a mucoid strain of *P*. *aeruginosa* under varying atmospheric conditions reflecting the range of microenvironments present in the CF lung and to characterise the interactions occurring between the two strains under hypercapnic conditions (10% CO_2_) using the co-culture system used previously in this context [28,29]. This study demonstrates that AGS, the potentially key but previously unrecognised ‘hidden pathogens’ in CF airway infection [25] can co-exist and thrive together with the mucoid phenotype of the most common CF pathogen, *P*. *aeruginosa* under a wide range of atmospheric conditions and can influence the responses of the latter to enhance its virulence potential. These findings add towards our understanding of the undoubtedly complex microbial dynamics of CF airway infections.

## Materials and methods

### Bacterial strains and culture conditions

*P*. *aeruginosa* strain DWW2 with a mucoid phenotype and from CF sputum (29) was routinely grown on LB agar (Invitrogen, United Kingdom) at 37°C. AGS (*Streptococcus anginosus*) strain 3a from CF sputum during exacerbation (33) was maintained at 37°C on blood agar (Blood Agar Base no.2, Oxoid, United Kingdom) containing 6% (vol/vol) defibrinated horse blood in an anoxic atmosphere (80% nitrogen, 10% hydrogen and 10% carbon dioxide). Liquid cultures for inoculating filters for biofilm growth were in Todd Hewitt Broth (Oxoid, United Kingdom) supplemented with 0.5% Yeast Extract (Becton Dickinson & Co., USA) (THY) incubated under normoxic conditions (in air) at 37°C with agitation (strain DWW2) or incubated under anoxic conditions (strain 3a).

For selection of DWW2 from co-cultures bacterial mixtures were plated onto Pseudomonas Isolation Agar (PIA) (Difco Laboratories, Becton Dickinson and Co., USA) and incubated under normoxic conditions at 37°C. For selection of 3a from co-cultures bacterial mixtures were plated onto a semi-selective agar (NAS) containing 1.1 gm per litre sulfamethazine and 37.5 mg / L nalidixic acid [33].

### Biofilm modelling

Static biofilms were grown on 47mm diameter, 0.22 μm pore nitrocellulose filters (Merck Millipore Limited, Ireland) placed on THY agar as previously described [34]. Replicate filters were inoculated with 100μl THY containing approximately 1.0 x 10^5^ colony forming units (CFU) of DWW2 or 3a either singly or in combination (co-cultures) and incubated at 37°C for 24h, under the conditions described for each of the experiments below. For growing biofilms containing DWW2 under anoxic conditions the THY agar was supplemented with 50 mM potassium nitrate. Depending on the aim of the experiment the biofilm incubation time points examined were 24 h, 48 h and 72 h.

After incubation the filters with biofilms were placed in 10mls of cold THY broth, vortexed for 1 min followed by scraping with a sterile disposable loop and finally vortexing for a further 30 s before serial dilution in THY broth and plating onto PIA for DWW2 counts and onto NAS for 3a counts. Data is given as the total CFU obtained per biofilm.

### Expression of pyocyanin by *P*. *aeruginosa* strain DWW2 in mono- and co-culture biofilms

Biofilms were cultured, harvested and processed for pyocyanin determinations as described previously [28,35]. Briefly, biofilms grown in 10% CO_2_ in monoculture or in co-culture with *S*. *anginosus* strain 3a were resuspended in 10mls THY broth, centrifuged at 2,700 g for 10 mins at 4°C, and supernatants harvested and filter sterilised by passing through a 0.22 μm filter. Pyocyanin was determined using the colorimetric assay described by Essar *et al* [36].

### Virulence in wax moth larvae (*Galleria mellonella*)

DWW2 and 3a were grown in THY broth as described above and washed 3 times in phosphate buffered saline (PBS). Bacteria were resuspended in PBS to give 4 x 10^7^ CFU/ml. 10 μl (= 4 x10^5^ CFU) were injected into the hemocoel via the last left proleg of *G*. *mellonella* larvae weighing 250–350 mg (Livefood UK Ltd, United Kingdom) as described previously [28]. After injection the larvae were incubated at 37°C in Petri dishes lined with filter paper. The numbers of viable larvae were recorded at 24 h and 48 h after injection. Larvae were considered dead when they displayed no movement in response to touch. Ten larvae were in each treatment group and experiments were repeated 3 times.

### Statistical analyses

Data are presented as mean ± standard deviation (SD) as a measure of data spread and were tested for normality by the Shapiro-Wilks test. Pairwise comparisons were by the 2-tailed Student’s t-test for parametric data or by the Mann-Whitney U test for non-parametric data. Comparisons between 3 or more groups were by one-way ANOVA with Tukey multiple comparisons post-hoc test or one-way ANOVA with Dunnet’s test for pairwise comparisons against a control. Data for larval survival experiments were analysed by a log-rank (Mantel-Cox) test for pairwise survival curve comparisons. A significance level of *p* < 0.05 was considered significant throughout.

## Results

### Analysis of mixed biofilm growth of *P*. *aeruginosa* strain DWW2 and *S*. *anginosus* strain 3a under normoxic, hypercapnic (10% CO_2_) and anoxic atmospheres

**Fig 1:**
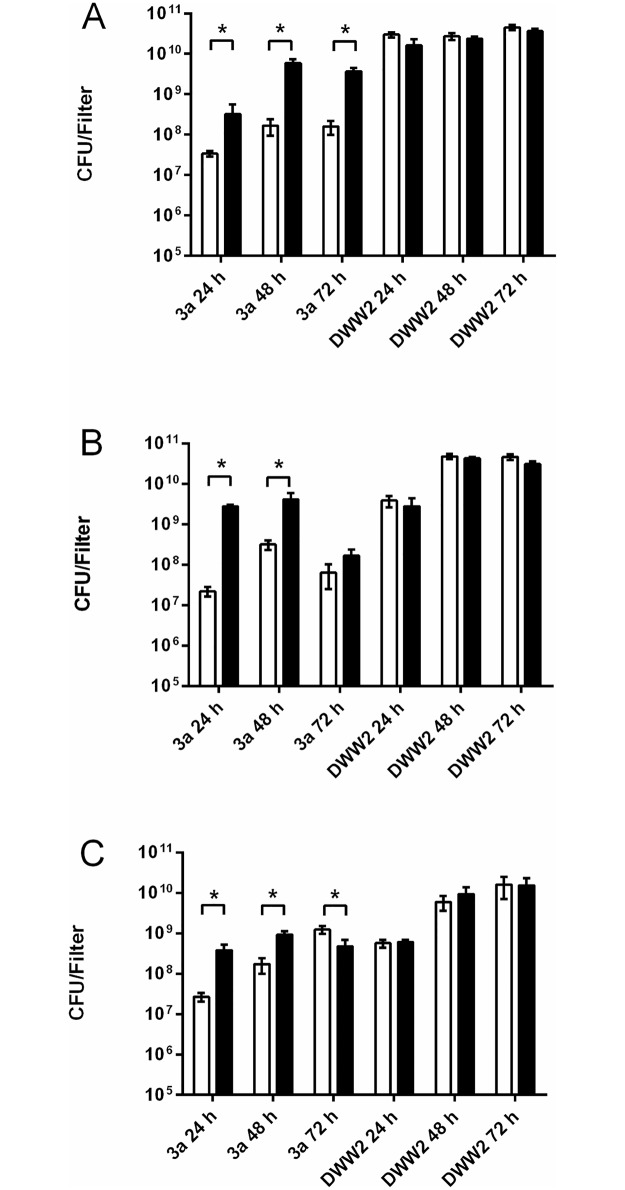
Quantitative bacteriology of biofilms. CFU/filter of monoculture (open bars) and co-culture (black bars) biofilms of *Pseudomonas aeruginosa* strain DWW2 and *Streptococcus anginosus* strain 3a at 24hrs, 48hrs and 72hrs grown in normoxic (air) (A), 10% CO_2_ (B) and anoxic (10% H_2_, 10% CO_2_, 80% N_2_) (C) environments. Data values are mean CFU per biofilm ± SD of 4 independent biofilms. Statistical analysis of bacterial counts obtained for strain 3a and DWW2 in mono-culture and in co-culture for each time point were by two-tailed Mann-Whitney U test: * = p<0.05.

The results of growing mono- and co-culture biofilms of strains DWW2 and 3a under different atmospheres are shown as total CFU per filter (Fig 1).

Both strains were able to grow singly and in combination under all test conditions with *S*. *anginosus* 3a increasing in numbers when in co-culture. The greatest increase in 3a counts was for growth in 10% CO_2_ between mono- (2.21 x 10^7^ ± 5.92 x 10^6^ CFU) and co-culture (2.8 x10^9^ ± 2.36 x 10^8^ CFU) biofilms at 24 h (a 127-fold increase). Under most atmospheric conditions tested there was an approximate 1–2 log_10_ increase in streptococcal numbers between monoculture and co-culture with exceptions at i) 72 h in 10% CO_2_ (6.33 x 10^7^ ± 3.84 x 10^7^ CFU in monoculture increasing to 1.68 x 10^8^ ± 7.35 x 10^7^ CFU in co-culture ≅ 2.65 fold increase) following a marked drop in counts between 48 h (4.09 x 10^9^ ± 1.92 x 10^9^ CFU) and 72 h (1.68 x 10^8^ ± 7.35 x 10^7^ CFU) when in co-culture, and ii) 72 h under anoxic conditions (1.24 x 10^9^ ± 2.74 x 10^8^ CFU in monoculture decreasing to 4.75 x 10^8^ ± 2.13 x 10^8^ CFU in co-culture). In contrast the numbers of DWW2 remained approximately the same in monoculture and co-culture with the highest numbers obtained in 10% CO_2_ at 48 h (4.83 x 10^10^ ± 7.51 x 10^9^ CFU in monoculture vs. 4.27 x 10^10^ ± 4.24 x 10^9^ CFU in co-culture).

### Progressive conversion of DWW2 to a Non-Mucoid (DWW2-NM) colony phenotype and development of localised variation in pyocyanin expression within DWW2 and DWW2+3a biofilms

**Fig 2:**
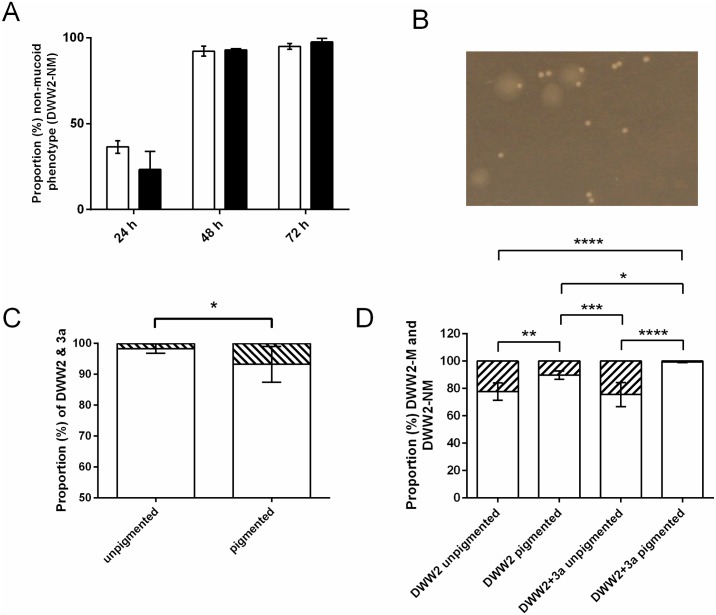
A non-mucoid colony phenotype of *P*. *aeruginosa* strain DWW2 (DWW2-NM) and higher numbers of *S*. *anginosus* strain 3a are associated with pigment (pyocyanin) production in co-culture biofilms. **(A)** The proportion (percentage) of the non-mucoid *P*. *aeruginosa* colony phenotype (DWW2-NM) increases over 72hrs in 10% CO_2_ in mono-culture (white bars) and in co-culture (black bars). Data values are mean and standard deviation of 4 whole independent biofilms in each case. **(B)** Larger mucoid (DWW2-M) and smaller, non-mucoid (DWW2-NM) colony phenotypes (derived from strain DWW2 48 h biofilms) cultured on PIA. **(C)** The proportions (percentages) of DWW2 (white bar) and 3a (black bar) in the sampled green pigmented and non-pigmented areas of co-culture biofilms at 48 h showing an increased proportion of 3a associated with pigment production. Data values are mean CFU per sample ± SD of 8 samples. Statistical analysis comparing bacterial counts obtained for strain 3a and DWW2 in pigmented and non-pigmented areas were by two-tailed Mann-Whitney U test: * = p<0.05. **(D)** The presence of *S*. *anginosus* 3a stimulates an increased conversion of DWW2 to the non-mucoid phenotype (DWW2-NM). The proportion (percentage) of mucoid (DWW2-M) (shaded bars) and non-mucoid (DWW2-NM) (white bars) colony phenotypes arising in non-pigmented and pigmented areas of mono- (DWW2) and co-culture (DWW2+3a) biofilms after 48 h incubation in 10% CO_2_. Data values are shown as mean and SD of 7 samples from pigmented and non-pigmented areas. Statistical analysis was by one-way ANOVA for parametric data with Tukey multiple comparison test (* = p<0.05; ** = p<0.01; *** = p<0.001; **** = p<0.0001).

**Fig 3:**
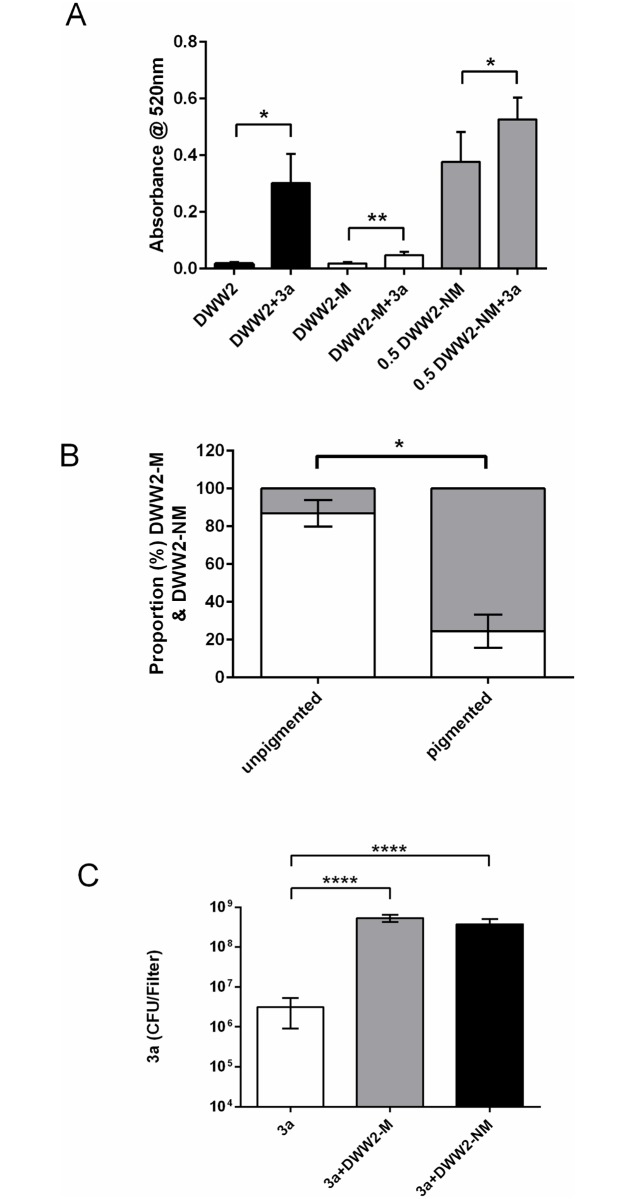
Examples of biofilms at 24 h, 48 h and 72 h. Examples of biofilms at the timepoints examined during these experiments are shown for i) monocultures of DWW2 (Row a), DWW2-M (Row c) and DWW2-NM (Row e), ii) co-cultures of 3a + DWW2 (Row b), 3a + DWW2-M (Row d) and 3a + DWW2-NM (Row f).

During biofilm development in 10% CO_2_ the proportion of non-mucoid colonies of DWW2 isolated on PIA was seen to increase dramatically between 24 h and 48 h in both mono- and co-culture with this phenotype comprising 94.73 ± 1.81% of the total DWW2 count in monoculture and 97.4 ± 2.1% in co-culture by 72 h (Fig 2A). Examples of DWW2 biofilm-derived non-mucoid and mucoid colonies were collected for further investigation in the biofilm model. In this paper the original mucoid *P*. *aeruginosa* CF strain is designated DWW2 and the mucoid and non-mucoid colony phenotypes isolated from DWW2 biofilms are designated DWW2-M and DWW2-NM, respectively. Concurrently, during biofilm incubation areas of varying intensities of pyocyanin production were observed to develop between 48 h and 72 h within individual DWW2 biofilms whether in monoculture (Fig 3a) or in co-culture with 3a (Fig 3b) in 10% CO_2_ (Fig 2D).

In order to compare the proportions of DWW2 and 3a in the high (pigmented) and low (non-pigmented) pyocyanin expressing locations within co-culture biofilms, replicate pigmented and non-pigmented zones were sampled using a 5 μl sterile disposable plastic bacteriological loop and the material collected transferred to 1ml of sterile PBS. After thorough resuspension by rotary agitation for 1 min the suspensions were serially diluted and spread onto PIA for DWW2 counts and onto NAS for 3a counts, expressed as a percentage of the total CFU. The proportion of DWW2 and 3a colonies in the high pyocyanin expressing (pigmented) and low pyocyanin expressing (non-pigmented) areas of co-culture biofilms at 48 h incubation are shown in Fig 2C.

The most obvious features of these data are the higher proportions of 3a in the high pyocyanin expressing, pigmented areas of the biofilms than in the non-pigmented areas: At 48 h strain 3a comprised 6.8 ± 5.81% of the biofilm in the pigmented areas (mean strain 3a count from 8 replicate samples = 2.65 x 10^7^ ± 1.84 x 10^7^ CFU vs. mean DWW2 count = 4.54 x 10^8^ ± 2.02 x 10^8^) compared with 1.69 ± 1.57% in the non-pigmented areas (mean strain 3a count from 8 replicate samples = 5.12 x 10^6^ ± 4.79 x 10^6^ CFU vs. mean DWW2 count = 2.83 x 10^8^ ± 1.72 x 10^8^) (p < 0.05) (Fig 2C).

### Effect of DWW2/3a biofilm co-culture on the conversion of DWW2 to the DWW2-NM phenotype

Although the data shown in Fig 2A showed no significant difference between the proportions of non-mucoid colonies arising in whole mono- and co-culture biofilms at the three timepoints sampled, the relative proportions of DWW2-NM arising in the pigmented and in the non-pigmented areas that were observable at approximately 48 h was investigated to determine whether the presence of *S*. *anginosus* strain 3a affected the degree of conversion of strain DWW2 to the DWW2-NM phenotype (Fig 2D). Briefly, replicate samples of pigmented and non-pigmented regions were obtained with a 5 μl disposable plastic bacteriological loop. The samples were processed and analysed for mucoid and non-mucoid colonies on PIA as described above. A significantly greater proportion of DWW2-NM colonies were present in the pigmented areas at 48 h (99.47 ± 0.55%) than were present in the non-pigmented areas (75.5 ± 8.8%) when in co-culture (p < 0.001). Further, there was a significantly higher proportion of DWW2-NM present in the pigmented areas of co-culture biofilms (99.47 ± 0.55%) than in the pigmented areas when in monoculture (89.68 ± 3.10%) (p < 0.05) indicating that the presence of *S*. *anginosus* strain 3a caused an increase conversion to the DWW2-NM phenotype. Subculturing DWW2-M and DWW2-NM colonies on PIA in 10% CO_2_ for 48 h demonstrated strong pyocyanin expression by DWW2-NM (Fig 3e) compared with DWW2-M (Fig 3c).

### Effect of co-culture with 3a on the expression of pyocyanin by DWW2, DWW2-M and DWW2-NM

**Fig 4:**
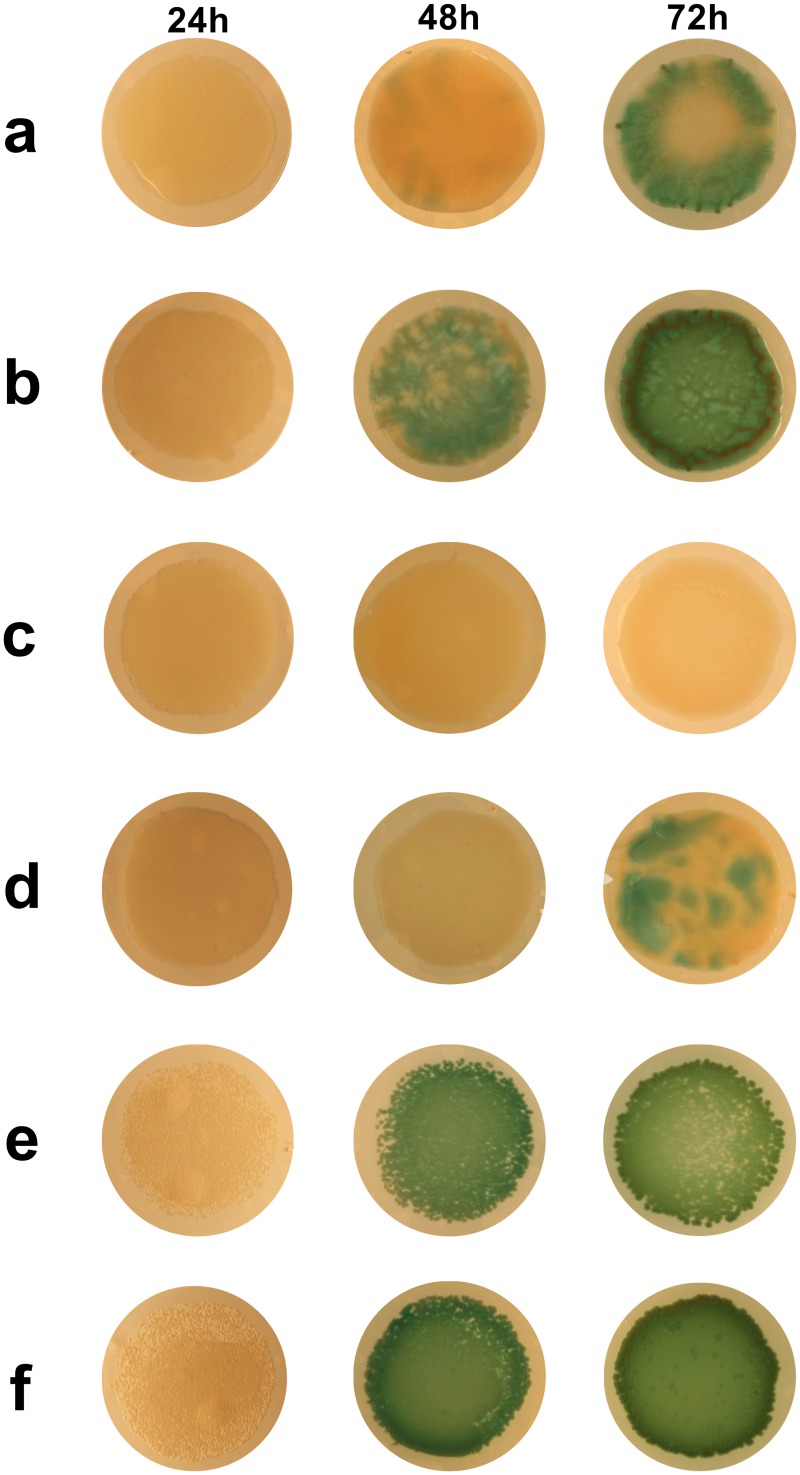
Interactions of colony phenotypes DWW2, DWW2-M and DWW2-NM with *S*. *anginosus* strain 3a. (A) Co-culture with 3a results in increased pyocyanin expression by the original strain DWW2 (black bars) (n = 3 independent biofilms at 48 h) and by the mucoid (DWW2-M) (white bars) (n = 6 independent biofilms at 72 h) and non-mucoid (DWW2-NM) (grey bars) (n = 6 independent biofilms at 48 h) biofilm-derived colony phenotypes. Extracted pyocyanin from DWW2-NM and DWW2-NM+3a were assayed at x0.5 original concentration to enable accurate determinations. Data values are mean and SD. Statistical analysis was by 2-tailed Student T-test assuming unequal variances (* = p <0.05; ** = p<0.01). (B) Co-culture of DWW2-M and 3a gives rise again to pigmented and non-pigmented areas with the non-mucoid phenotype (DWW2-NM) predominant in the pigmented areas (DWW2-NM biofilms in mono-culture or co-culture did not give rise to the DWW2-M phenotype). Data values are means and SD (n = 4 independent biofilms at 72 h). Statistical analysis of the data was by two-tailed Mann-Whitney U test. (C) Co-culturing 3a with either DWW2-M or DWW2-NM results in significantly increased numbers of 3a as observed for co-culture between 3a and the original mucoid strain DWW2. Data values are means and SD (n = 6 independent biofilms at 24 h). Statistical analysis of the data was by one-way ANOVA with Dunnet’s multiple comparison test between 3a in mono-culture (control) and co-cultures (**** = p<0.0001).

The effect of the presence of *S*. *anginosus* strain 3a on pyocyanin expression by strain DWW2 and by DWW2-M and DWW2-NM was investigated. The data are shown in Fig 4A.

Co-culture with *S*. *anginosus* strain 3a significantly increased the expression of pyocyanin by DWW2 (mean fold increase = 17.70; p < 0.05). Similarly, although low levels of pyocyanin were detected for DWW2-M, increased pyocyanin expression was also detected when in co-culture with 3a (mean fold increase = 2.6, p< 0.01). High levels of pyocyanin were expressed in both monoculture and co-culture biofilms with DWW2-NM requiring dilution of the extracted pigment to enable quantitation. The data measured at 0.5 original strength of the DWW2-NM biofilm extracts show that co-culture with 3a resulted in increased pyocyanin expression (1.4 fold increase, p<0.05). The non-pigmented and pigmented areas seen in DWW2 mono- and co-culture biofilms were also observed in DWW2-M + 3a co-culture biofilms but not for DWW2-NM + 3a due to over production of pyocyanin by DWW2-NM (Fig 3e and 3f). The proportions of mucoid and non-mucoid types present in the pigmented and non-pigmented areas of the DWW2-M + 3a biofilms again show that a high proportion of pyocyanin producing non-mucoid types are present in the pigmented areas (75.5 ± 8.8%) in contrast to the non-pigmented (13.1 ± 7.01%) (p<0.05) (Fig 4b).

### Effect of DWW2-M and DWW2-NM on *S*. *anginosus* strain 3a numbers when in co-culture

Colony counts of strain 3a were determined from 24 h biofilms in monoculture and in co-culture with DWW2-M and DWW2-NM. The aim was to determine whether co-culturing resulted in enhanced growth of the streptococcus as had been observed between 3a and the original strain DWW2. Significant increases in the numbers of 3a were observed in co-culture biofilms with DWW2-M (5.35 x 10^8^ ± 1.08 x10^8^ CFU) and with DWW2-NM (3.79 x 10^8^ ± 1.26 x 10^8^ CFU) compared with 3a in monoculture (3.11 x10^6^ ± 2.21 x10^6^ CFU) (p<0.0001) (Fig 4c).

### Effect of mono- and co-culture infections with 3a, DWW2, DWW2-M and DWW2-NM on *G*. *mellonella* survival

**Fig 5:**
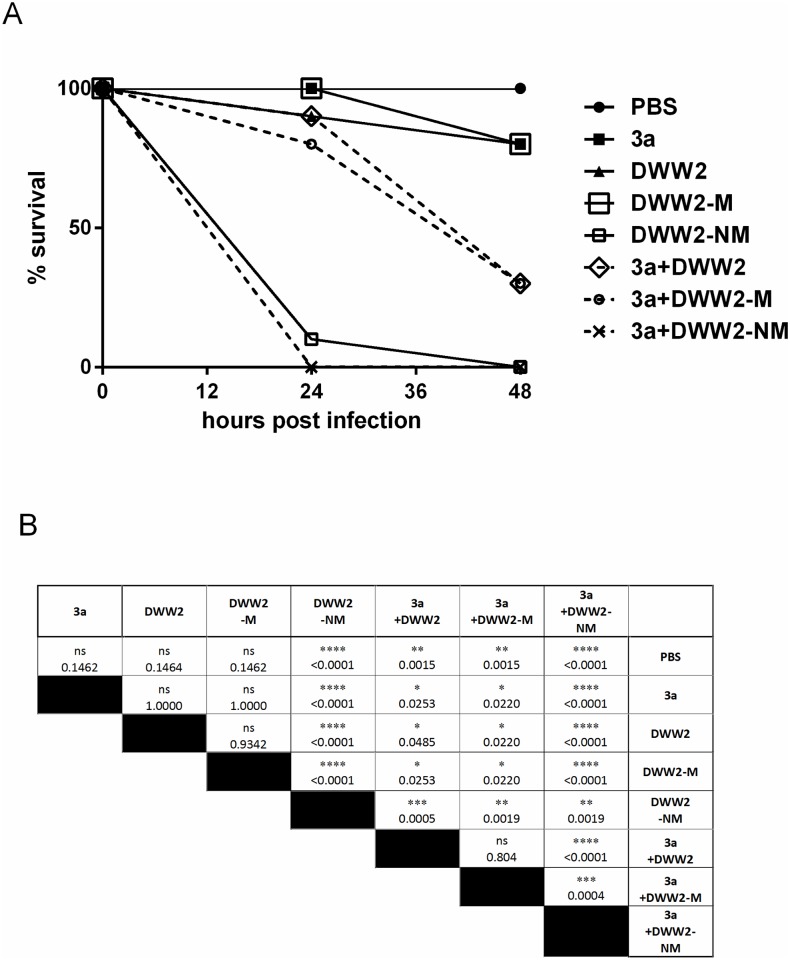
Potentiated virulence in the *Galleria mellonella* infection model by the non-mucoid phenotype (DWW2-NM) and co-infection with *P*. *aeruginosa* and *S*. *anginosus* strain 3a. **A)** Graph of percent larval survival post-infection at 24 h and 48 h. Ten larvae were in each infection group and in the control (PBS only) group. The results shown are from Experiment 1 of three repeated experiments (see also S1 and S2 Figs). **B)** Matrix of pairwise comparisons of survival curves between infection regimes obtained in Experiment 1 by the log-rank (Mantel-Cox) test. ns = not significant, p>0.05; * p<0.05; ** p<0.01; *** p<0.001; **** p<0.0001 (see also S1 and S2 Figs).

The *G*. *mellonella* larval survival data at 24 h and 48 h post-inoculation with strains either singly or in combination are shown in Fig 5A. A matrix of pairwise comparisons between the survival curves for each infection regime are shown in Fig 5B. The results of two replicate experiments are shown S1 and S2 Figs.

After 24 h incubation DWW2-NM (as mono-infection or co-infection with 3a) was clearly the most virulent infecting agent resulting in significantly less larval survival (10% survival for mono-infection and 0% survival for co-infection) compared with all other infection regimes (p < 0.001). Significant decrease in larval survival was also observed between DWW2-NM alone and DWW2-NM+3a co-infection (p<0.01). Co-infection of DWW2 or DWW2-M with 3a also resulted in significantly increased virulence than when mono-infecting (p<0.05). No significant differences in larval survival were observed between the other experimental infections at 24 h (p > 0.05). This overall pattern of relative virulence was also observed in the replicate experiments (Expts 2 & 3) (S1 and S2 Figs) with the exceptions of mono-infections with DWW2 and DWW2-M which were relatively variable between experiments.

## Discussion

The presence of a diverse and active microbial community within the infected CF airway has been recognised for some time [15,16,17,37,38,39]. However, interactions between community members in these spatially and environmentally heterogeneous niches need to be resolved if the relationship between microbiome dynamics and CF pathogenesis is to be understood [22,27,40,41]. In the context of CF airway infection, studies focussing on interactions between bacteria should, where possible, address the environmental variations and fluctuations to which the microorganisms are exposed. In previous studies undertaken in our laboratory AGS were reported as able to co-exist with *P*. *aeruginosa* [28,29]. However those studies were limited to non-mucoid phenotypes of the Liverpool Epidemic strain (LES) and therefore the present study undertook to investigate the interactive behaviour of an alginate over expressing, mucoid *P*.*aeruginosa* strain of the phenotype commonly isolated from CF airways [42,43]. Adaptation of infecting *P*. *aeruginosa* strains by switching to a mucoid phenotype is one of the most potentially clinically significant responses of this pathogen during chronic infection of the CF airway and therefore deserving of more detailed study including characterisation of its interactions with other bacteria such as the AGS which have been highlighted as potentially significant in CF pathogenesis. Here we investigated the potential for co-culture biofilm growth and interaction between *Pseudomonas aeruginosa* mucoid CF strain DWW2 and a member of the Anginosus species group of streptococci (AGS), *Streptococcus anginosus* CF strain 3a, in an *in-vitro* biofilm model. The experiments undertaken here demonstrated that these bacteria were able to co-exist and multiply in atmospheric conditions reflecting the varied environments present in the CF airways including normoxic, hypercapnic (10% CO_2_) and anoxic conditions [13,44,45]. That *P*. *aeruginosa* can undergo anoxic growth by denitrification in the presence of physiological levels of nitrate has been previously demonstrated [46] indicating that anaerobic respiration by this pathogen could occur within the CF lung. However, to our knowledge the potential for mixed growth of a mucoid *P*. *aeruginosa* strain with other bacterial species in a range of environmental atmospheric conditions has not been investigated previously. As seen with LES/AGS biofilms, co-culture here of DWW2 and 3a over 48 h resulted in significantly higher numbers of the streptococcus than were observed in monocultures. Together these studies demonstrate that the AGS streptococci are able to co-exist with *P*. *aeruginosa* despite the presence of pyocyanin with its potential antimicrobial activity [47]. In the present study biofilms were incubated for up to 72 h and a decrease in streptococcal counts was observed to occur between 48 h and 72 h for co-cultures in 10% CO_2_. In this static (closed system) biofilm model nutrient depletion and accumulation of toxic metabolic products that possibly include effects due to pyocyanin may well be sources of stress resulting in the observed reduction in cell numbers [48]. We conclude that the current model system is reliable for up to 48 h incubation within which useful observations about these microbial partnerships are possible and that investigations into longer term mixed culture dynamics between these bacteria will ideally utilise an open continuous culture model system. In the present study the increased streptococcal numbers in co-culture biofilms may be of direct relevance to the microbial fluctuations that occur in CF airways; although the study by Stressmann et al [49] did not reveal a correlation between increased overall bacterial density in CF airways and occurrence of exacerbation these authors emphasised that the absence of changes in total microbial burden did not preclude the possibility of clinically significant increases occurring in individual, non-pseudomonal community members. In support of this caveat another longitudinal study of CF patients reported AGS proliferation to numerical dominance before onset and during exacerbations [25]. In the present study the stimulation of AGS to proliferate by a mucoid *P*. *aeruginosa* strain demonstrates that these taxa are well suited to co-exist in close partnership albeit in an *in-vitro* model and may well constitute an important partnership within the CF airway during clinical stability and exacerbation.

More detailed examination of the interaction between DWW2 and 3a in a hypercapnic environment (10% CO_2_) demonstrated increased virulence factor (pyocyanin) expression by the pseudomonad when in co-culture as seen previously with an LES and AGS partnership and between wound strain PAO1 and streptococci from the oropharynx [27,28,29]. The results from this study also demonstrated that the increase in pyocyanin was not due to increased expression throughout the biofilm but to the development of discrete pigmented areas within the biofilm that were dominated by the high pyocyanin expressing, non-mucoid phenotype together with higher numbers of the streptococcus which in turn was associated with an increased conversion of strain DWW2 from mucoid to non-mucoid. Non-pigmented areas of the biofilm, *ie* without visible pyocyanin expression, were dominated by the mucoid phenotype accompanied by significantly lower numbers of streptococci. Conversion of CF infecting strains of *P*. *aeruginosa* to the alginate overproducing, mucoid phenotype accompanied by auxotrophy together with loss of both motility and virulence are adaptations to the stresses of the CF airway and widely considered to be markers of transition to chronic infection [50,51,52,53]. Mucoidy is caused by mutations in genes (*muc A*, *muc B* or *muc D*) of the alginate biosynthesis operon through loss of the negative regulator effects of these gene products on alginate synthesis [54,55]. The advantages of mucoidy in the context of CF are enhanced biofilm formation giving protection from phagocytic clearance and from antimicrobial therapy resulting in a predominance of this phenotype in CF (see Hauser et al [56] for a review of the literature). However the significance of the non-mucoid revertants in the dynamics of CF is less clear but may also be important over time and in disease development. Reversion to the non-mucoid phenotype occurs *in-vivo* and both non-mucoid and mucoid phenotypes are commonly present in CF sputa [45,57,58]. Examination of explanted lungs from CF patients has shown non-mucoid and planktonic cells as well as mucoid cells in the conductive zone of the CF airways together with large numbers of PMNs suggesting that the conductive zone may act as a bacterial reservoir where the bacteria are organized in mucoid biofilms [45]. In a study using an agar bead murine model of infection, sequential isolates of *P aeruginosa* from CF airways showed that some but not all strains became mucoid and that both mucoid and non-mucoid phenotypes of *P*.*aeruginosa* showed a similar capacity of persistence in the mouse lung. In the same report a study of CF patients showed that the majority were persistently infected by non-mucoid variants which on isolation were able to persist in mouse airways. Non-mucoid cells were able to form macrocolonies outside the agarose beads within the bronchial lumen and both mucoid and non-mucoid types provoked extensive inflammatory responses in the bronchial lumen and thickened alveolar septa [59]. It is known that adaption of *P aeruginosa* to persist in CF airways in chronic infections occurs through loss of function mutations [53,60]. Using the same murine agarose bead model of chronic airways infection and by tracking patho-adaptive, loss of function mutations Briaconi et al [61] identified genes whose inactivation increased colonization and persistence in chronic airways infection. Interestingly these authors did not detect mutants in those functions associated with alginate biosynthesis that resulted in mucoidy and speculated that the mucoid phenotype appears relatively late in the chronic infection and might not be critical for the establishment and earlier stages of chronic infection. Fothergill et al [62] analysed sputa from chronically infected CF patients at the beginning, during and end of exacerbation and in all samples the majority of isolates were non-mucoid rather than exhibiting the mucoid phenotype commonly associated with chronic infection. These authors measured a decrease in the numbers of the non-mucoid type later on during the exacerbation but pointed out that pyocyanin overproduction associated with the numerically dominant non-mucoid phenotype could potentially increase oxidative effects in CF patients. CF sputum levels of pyocyanin have been shown to correlate with disease severity [63]. The results of measuring pathogenicity in *Galleria mellonella* lend some support to this hypothesis with significantly greater larval killing with infection by DWW2-NM than with DWW2 or DWW2-M. If non-mucoidal variants of CF infecting *P*. *aeruginosa* do contribute to airway damage as Fothergill et al [62] suggest then the increased virulence seen here with DWW2-NM + 3a co-infections supports the idea that these interactions might be potentially clinically significant. That inter-strain variation exists amongst CF isolates is no doubt the case [64] and further studies need to be undertaken to determine how strain-variable are these interspecies dynamics. In this context Ryall et al [65] showed that mutation in mucA giving the mucoid phenotype can cause subtle changes in pyocyanin production depending upon the phase of growth of *P*.*aeruginosa*. These authors observed down-regulation of pyocyanin upon entry into early stationary phase but in contrast to wild type strains continued pyocyanin production when in prolonged stationary phase, a finding that may have important consequences in vivo during chronic infection under non-growing conditions.

In summary the results of the present study demonstrate that an AGS strain can benefit from living in proximity to a mucoid *P*. *aeruginosa* strain and its non-mucoidal revertants while significantly modulating the behaviour of the pseudomonad in ways that may be important to disease progress. As in our previously published studies of *P*. *aeruginosa* and AGS we chose to examine these interactions in 10% CO_2_ because this is particularly relevant to conditions in the cystic fibrosis lung where impaired gaseous exchange is thought to cause the increased levels of alveolar CO_2_ observed in the CF lung [66]. It will be of interest to extend these studies to determine the responses of these strains singly or in combination to a wider range of atmospheric conditions that may be encountered in this disease environment.

## Supporting information

S1 Dataset Data used in analyses. (XLSX)

S1 Fig Larval survival replicate experiment 1. (A) Graph of percent larval survival post-infection at 24 h and 48 h in replicate experiment 2. Ten larvae were in each infection group and in the control (PBS only) group. (B) Matrix of pairwise comparisons of survival curves between infection regimes obtained in Experiment 2 by the log-rank (Mantel-Cox) test.
(TIF)

S2 Fig Larval survival replicate experiment 2. (A) Graph of percent larval survival post-infection at 24 h and 48 h in replicate experiment 3. Ten larvae were in each infection group and in the control (PBS only) group. (B) Matrix of pairwise comparisons of survival curves between infection regimes obtained in Experiment 3 by the log-rank (Mantel-Cox) test.
(TIF)
