# Correction: Modulation of behaviour and virulence of a high alginate expressing *Pseudomonas aeruginosa* strain from cystic fibrosis by oral commensal bacterium *Streptococcus anginosus*

**DOI:** 10.1371/journal.pone.0176577

**Published:** 2017-04-20

**Authors:** Richard D. Waite, Muhammad R. Qureshi, Robert A. Whiley

The images for Figs 3 and 4 are incorrectly switched. The image that appears as Fig 3 should be Fig 4, and the image that appears as Fig 4 should be Fig 3. The figure captions appear in the correct order.

**Fig 3:**
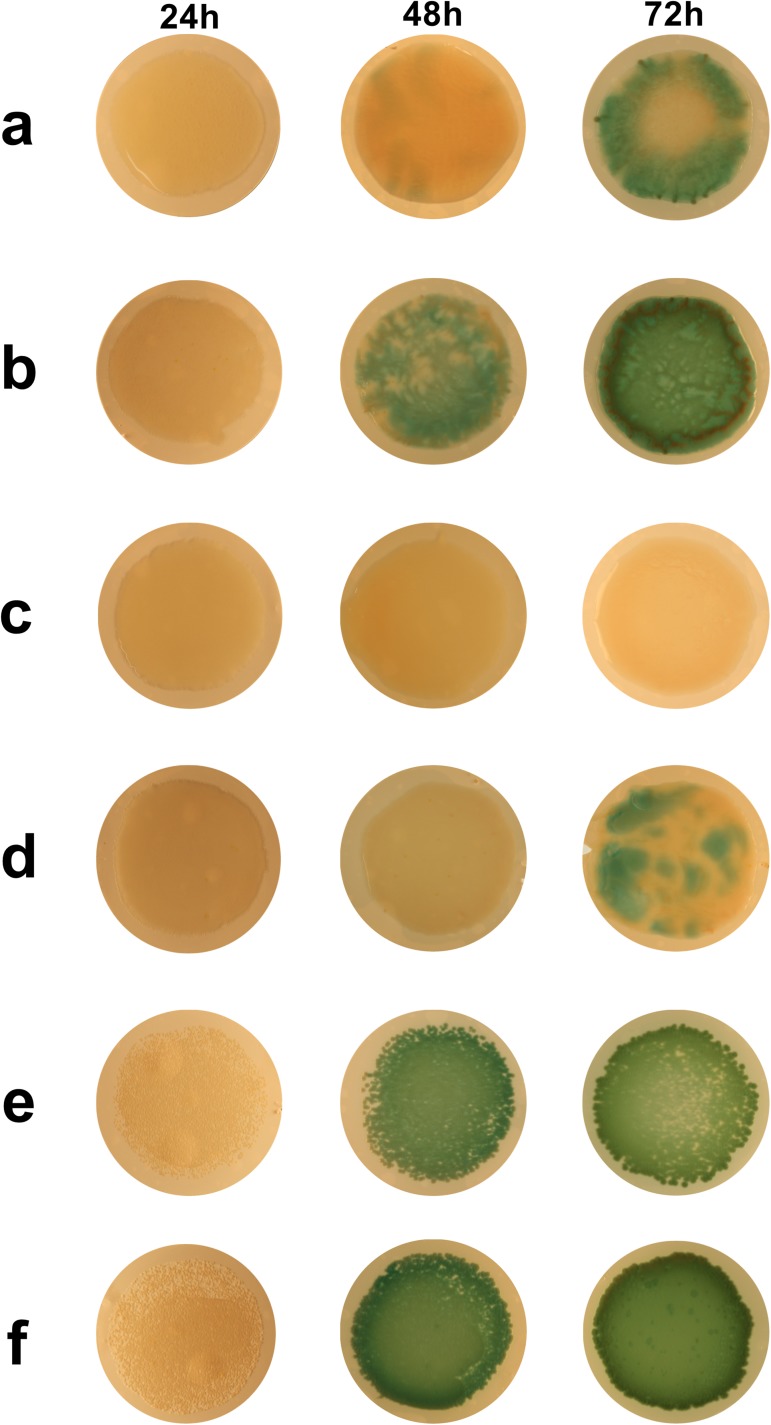
Examples of biofilms at 24 h, 48 h and 72 h. Examples of biofilms at the timepoints examined during these experiments are shown for i) monocultures of DWW2 (Row a), DWW2-M (Row c) and DWW2-NM (Row e), ii) co-cultures of 3a + DWW2 (Row b), 3a + DWW2-M (Row d) and 3a + DWW2-NM (Row f).

**Fig 4:**
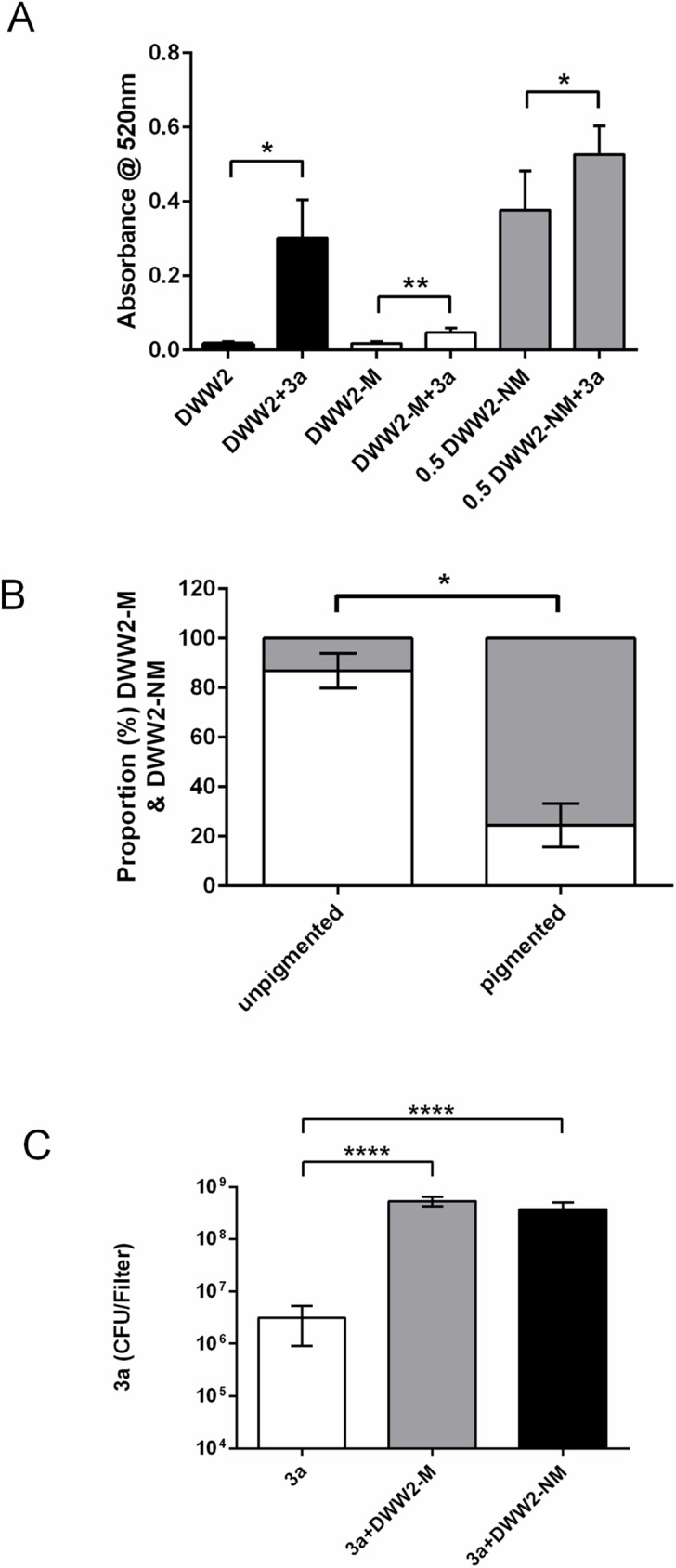
Interactions of colony phenotypes DWW2, DWW2-M and DWW2-NM with *S. anginosus* strain 3a. (A) Co-culture with 3a results in increased pyocyanin expression by the original strain DWW2 (black bars) (n = 3 independent biofilms at 48 h) and by the mucoid (DWW2-M) (white bars) (n = 6 independent biofilms at 72 h) and non-mucoid (DWW2-NM) (grey bars) (n = 6 independent biofilms at 48 h) biofilm-derived colony phenotypes. Extracted pyocyanin from DWW2-NM and DWW2-NM+3a were assayed at x0.5 original concentration to enable accurate determinations. Data values are mean and SD. Statistical analysis was by 2-tailed Student T-test assuming unequal variances (* = p <0.05; ** = p<0.01). (B) Co-culture of DWW2-M and 3a gives rise again to pigmented and non-pigmented areas with the non-mucoid phenotype (DWW2-NM) predominant in the pigmented areas (DWW2-NM biofilms in mono-culture or co-culture did not give rise to the DWW2-M phenotype). Data values are means and SD (n = 4 independent biofilms at 72 h). Statistical analysis of the data was by two-tailed Mann-Whitney U test. (C) Co-culturing 3a with either DWW2-M or DWW2-NM results in significantly increased numbers of 3a as observed for co-culture between 3a and the original mucoid strain DWW2. Data values are means and SD (n = 6 independent biofilms at 24 h). Statistical analysis of the data was by one-way ANOVA with Dunnet’s multiple comparison test between 3a in mono-culture (control) and co-cultures (**** = p<0.0001).
