# Supplementary figures and images for: Modulation of behaviour and virulence of a high alginate expressing Pseudomonas aeruginosa strain from cystic fibrosis by oral commensal bacterium Streptococcus anginosus
Source: PLoS One. 2017 Mar 16;12(3):e0173741. doi: 10.1371/journal.pone.0173741 (PMC5354419; doi:10.1371/journal.pone.0173741)

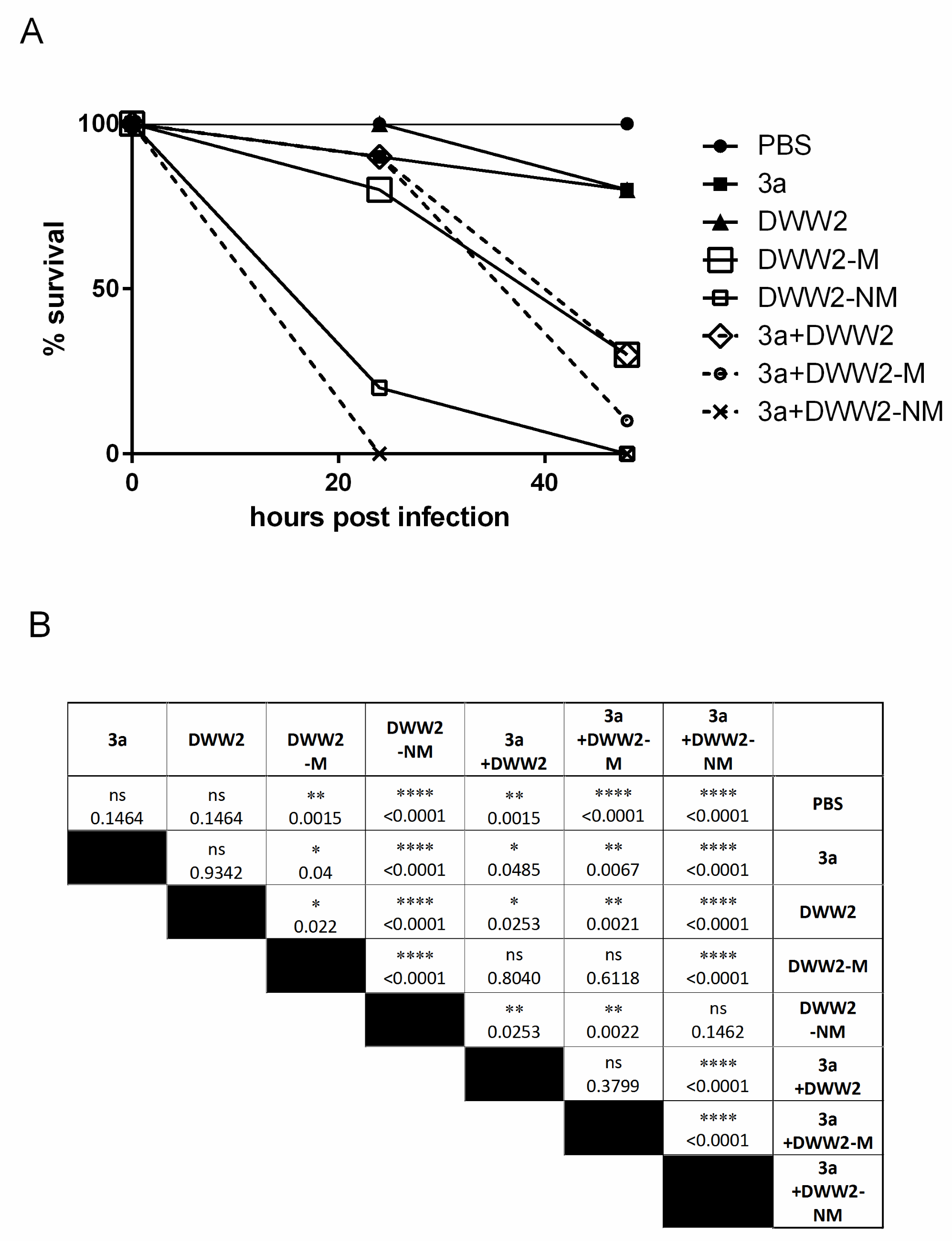

Supplement: S1 Fig — (A) Graph of percent larval survival post-infection at 24 h and 48 h in replicate experiment 2. Ten larvae were in each infection group and in the control (PBS only) group. (B) Matrix of pairwise comparisons of survival curves between infection regimes obtained in Experiment 2 by the log-rank (Mantel-Cox) test. (TIF) [file pone.0173741.s002.tif]

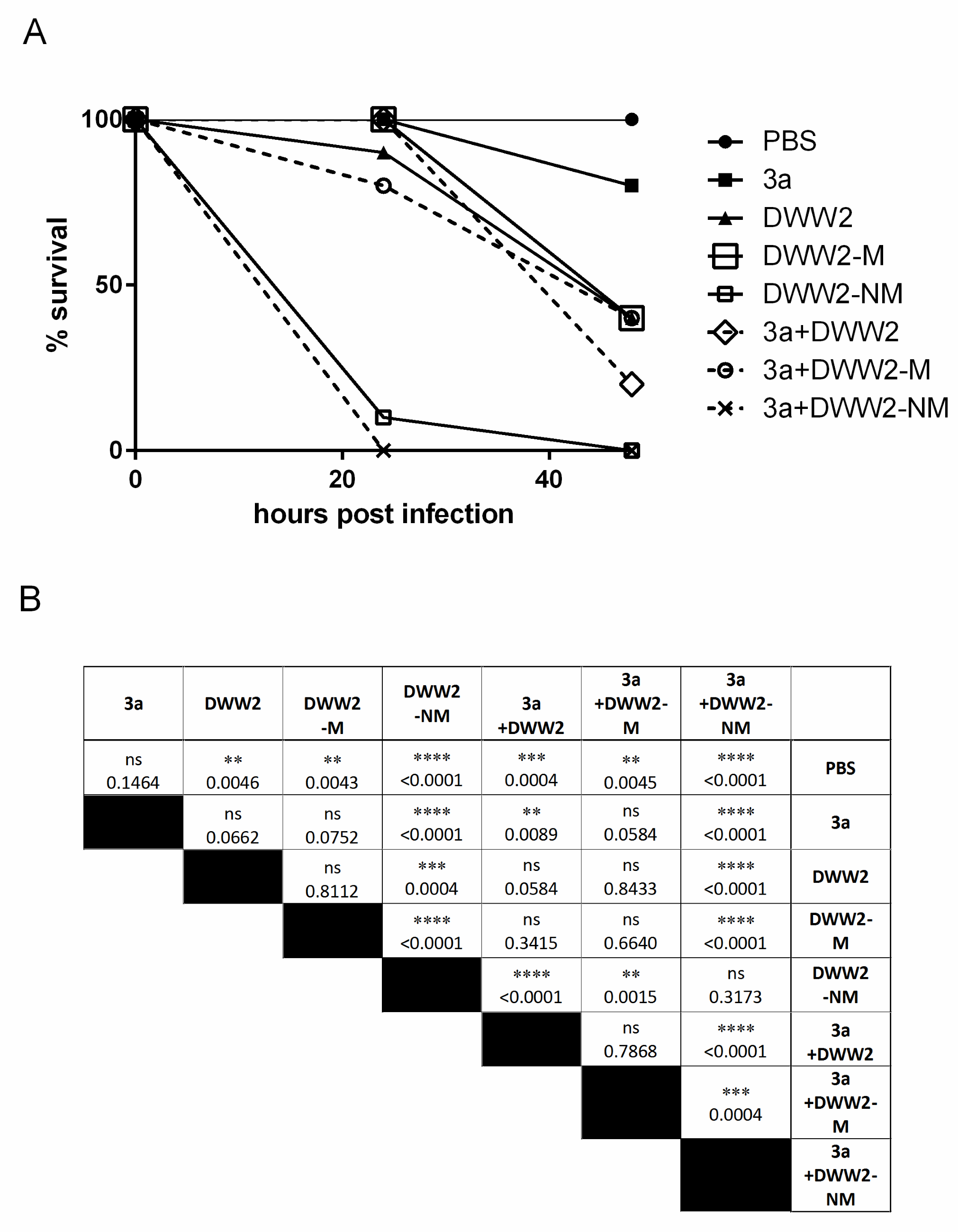

Supplement: S2 Fig — (A) Graph of percent larval survival post-infection at 24 h and 48 h in replicate experiment 3. Ten larvae were in each infection group and in the control (PBS only) group. (B) Matrix of pairwise comparisons of survival curves between infection regimes obtained in Experiment 3 by the log-rank (Mantel-Cox) test. (TIF) [file pone.0173741.s003.tif]
